# Unlocking the organic residues preserved in the corrosion from the Pewsey Hoard vessels

**DOI:** 10.1038/s41598-022-24400-5

**Published:** 2022-12-09

**Authors:** Luciana da Costa Carvalho, Richard Henry, James S. O. McCullagh, A. Mark Pollard

**Affiliations:** 1 School of Archaeology, University of Oxford, Oxford, UK; 2 Department of Archaeology, University of Reading, Reading, UK; 3 Chemistry Research Laboratory, Department of Chemistry, University of Oxford, Oxford, UK

**Keywords:** Analytical chemistry, Organic chemistry

## Abstract

The characterization of archaeological metal corrosion has traditionally been limited to the identification of inorganic compounds usually by X-ray diffraction (XRD), thought to result from the interaction between the metal object and the deposition environment. The discovery of a hoard of Late Roman copper-alloy vessels in Wiltshire, UK presented an unique opportunity to adopt a multi-analytical approach to characterize corrosion combining XRD with Fourier-transform infrared (FTIR) and gas chromatography with quadrupole time-of-flight mass spectrometry using a thermal separation probe (GC-QTOF-MS with TSP). This approach revealed organic compounds potentially historical preserved within crystalline inorganic matrices. It has been known for some time that ceramics can harbour organic residues, which provide crucial evidence about the use of these vessels in the past. Our results confirms that similar residues appear to survive in metal corrosion thus extending the potential for identification of biomaterials used in the past.

## Introduction

The identification of substances used in the past enhances our understanding of cultural practices and everyday life in the past^1^. Although the earliest analyses of visible archaeological residues were undertaken in the early twentieth century^2^, it was the coupling of chromatography with mass spectrometry 50 years later that enabled the chemical profiling of complex organic residues^3,4^.

The study of archaeological organic residues is complicated by chemical changes due to the processing of substances (e.g. mixing, cooking) in the past, their degradation during deposition, and contamination^5^. Notwithstanding these complexities, an increasing number of markers for specific substances are being reported in the literature (e.g.^4,6–18^). Currently, organic residues are mostly extracted from porous ceramic sherds^4^—their porosity believed to confer some protection against degradation and contamination in a way which is yet to be fully understood.

Conservators routinely find macro-organic remains such as fibres, insects, pollen and plant fragments “mineralized” by archaeological copper corrosion products, particularly from metal objects recovered from graves^19^. In copper alloy objects, preservation of organic residues may include encapsulation by inorganic salts^20^ aided by copper’s biocidal properties^21–24^ and the formation of metal–organic complexes^25^.

In a pilot study of bronze corrosion, Merriman et al*.*^26^ recovered pine resin and oil markers from the corrosion of a *patera*, a pan-like object found in ritual contexts throughout the Roman empire. The innovation of this study was the targeting of corrosion for organic residue analysis, as opposed to visually distinguishable residues (e.g.^21,27–30^). The researchers suggested that the organic compounds recovered had been encapsulated by inorganic compounds (although these were not identified as part of the study) as opposed to being trapped in the sample as metal–organic or organometallic complexes which would be harder to detect.

Indeed, corrosion experiments have demonstrated that copper ions readily react with organic and inorganic ligands in the same medium^31^ forming copper-organic complexes and characteristic inorganic phases. Moreover, the low solubility of copper-organic complexes in organic solvents^32^ and/or the presence of copper ions negatively impact the recovery of organic residues by typical gas chromatography with mass spectrometry protocols^33^. Thus the discovery of the Pewsey Hoard provided a unique opportunity to investigate archaeological corrosion from difference vessels with the same deposition context by incorporating techniques targeting both inorganic and organic compounds.

## The Pewsey Hoard

**Figure 1:**
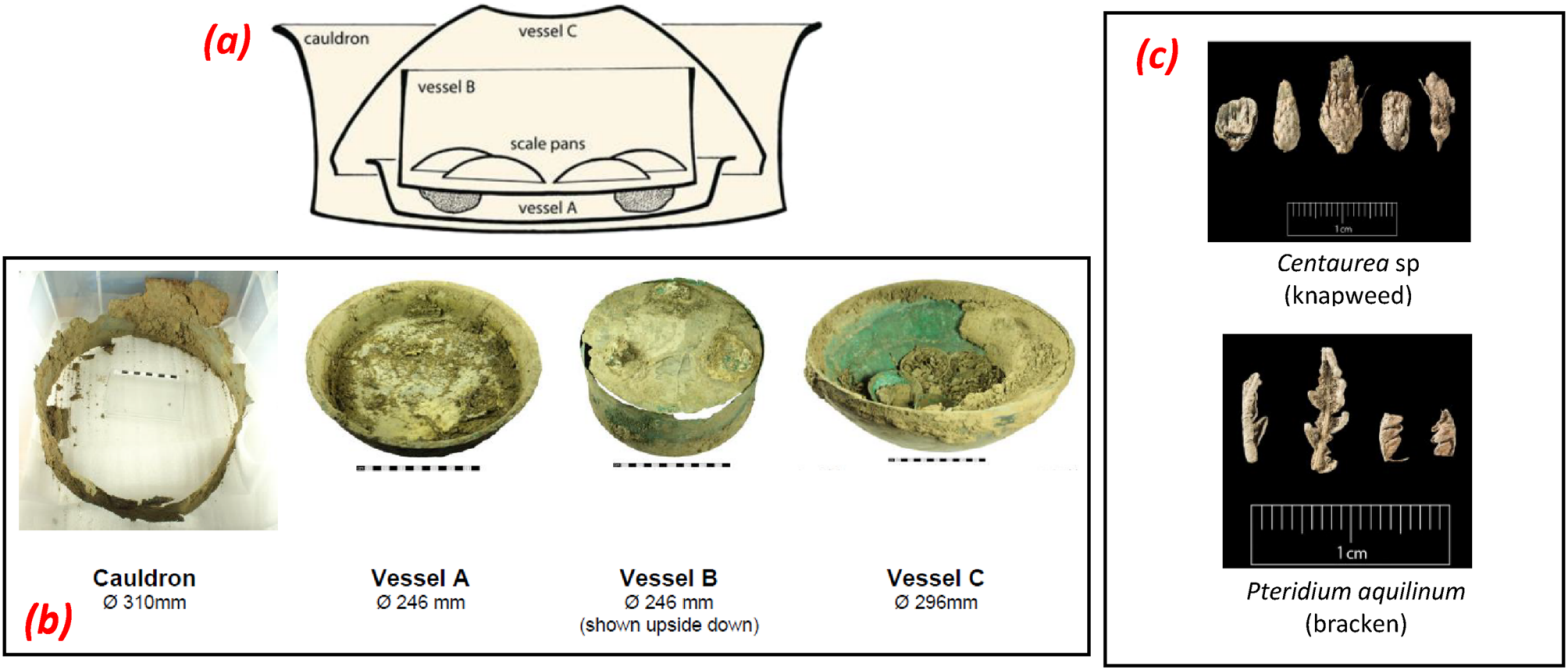
The Pewsey Hoard (**a**) graphic representation of the arrangement of the objects (**b**) the main vessels as delivered to Salisbury Museum (**c**) plant remains found in Vessel B. Source: https://finds.org.uk/database/artefacts/record/id/720549.

The Pewsey Hoard is a group of Late Roman copper-alloy vessels found by metal detectorists in 2014 in a field in the Vale of Pewsey, Wiltshire (UK)^34^. Excavated by the finders themselves, it consists of a large iron-rimmed copper-alloy cauldron (Fig. 1a,b) holding two bowls (Vessels A and C) and another vessel (Vessel B) containing four scale pans carefully packed with plants (Fig. 1c). Subsequent excavation of the area surrounding the findspot by Historic England concluded that the hoard was deposited in a pit, dug in an actively used landscape with no structures or ditches in its immediate vicinity^35^.

Although several Late Roman hoards containing cauldrons and bowls have been found in Britain, the Pewsey Hoard is unusual because of the inclusion of scale pans and plant packing material. The combination of objects of different functions points to the broad economic value of metal artefacts but the inclusion of the scale pans (possibly pairs given their similar weight and dimension), uncommon in a rural setting, suggests a symbolic intention^36^. Analysis of the various grassy woodland plant remains found “mineralized” in Vessel B due to a specific microclimate, indicates that the hoard was deposited in an arable field immediately after the cereal harvest, sometime within AD 345–405 period. The discovery, in 2020, of a Late Roman coin hoard containing of 160 silver coins^37^ scattered in the same field emphasizes the importance of the site within the wider landscape.

The corrosion covering the Pewsey Hoard vessels varied in colour and texture, sometimes within the same vessel (Table 1) suggesting exposure of the metal surface to different environments, prior and/or during deposition. Therefore, the sampling strategy adopted for this study was based on the following considerations:

- The closest corrosion layer to the metallic surface would be most likely to contain historic significant residues;
- Substances related to vessel use may be present in the interior corrosion but not in the exterior;
- The areas to be sampled should be those less likely to have been contaminated due to handling during excavation;
- A soil sample from within the hoard would act as blank/control.

**Table 1:** Samples collected from the Pewsey Hoard.

Vessel	Location	Colour	Texture
Cauldron rim	Interior	Blue/greenish	Hard
	Exterior	Blue/greenish	Hard
Vessel A	Interior	Green/brownish	Powdery
	Exterior	Dark brown/black	Hard
Vessel B	Interior (body/side)	Green/brownish	Powdery
	Interior (base/bottom)	Green/bluish	Hard
Other	Soil under vessel B	Brownish	Powdery with crystals

To target inorganic and organic phases within the same sample we adopted a multi-analytical approach for the characterization of the Pewsey Hoard corrosion comprised of:

- *Fourier-transform infrared (FTIR)* a popular technique with museum conservators for the identification of organic and inorganic materials;
- *Powder X-ray diffraction (XRD)* a technique used for the identification of crystalline compounds, commonly used for the identification of mineral phases in archaeological metal corrosion;
- *Gas chromatography with quadrupole time-of-flight mass spectrometry using a thermal separation probe (GC-QTOF-MS with TSP)* a relatively recent technique for the study of organic residues trapped in inorganic matrices such as soils^38^.

The aim of our study was to investigate if organic residues were preserved in association with mineral phases, consider if these residues could be of historical value and evaluate the analytical techniques.

## Results and discussion

### The composition of the cauldron rim interior corrosion appears to be influenced by the soil

Initially, the cauldron rim corrosion samples were analysed by FTIR in ATR mode but this technique resulted in a spectra with poorly-defined bands and baseline. In an attempt to obtain an improved spectra, the interior corrosion of the vessel was analysed using the KBr method, which is destructive. There wasn't enough exterior sample to analyse by this method.

**Figure 2:**
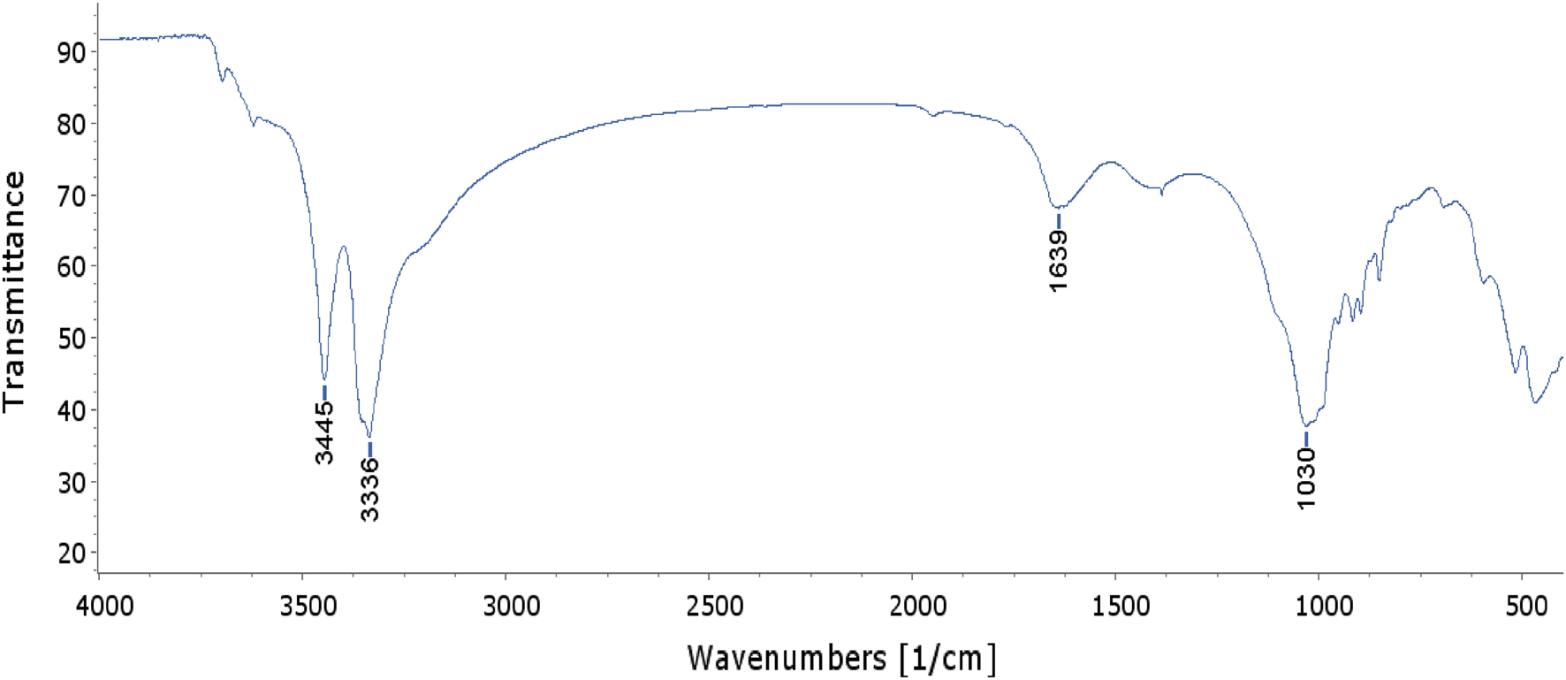
FTIR spectrum for the interior corrosion of the Cauldrom Rim.

Diagnostic bands in the FTIR spectra (Fig. 2) of the cauldron rim interior corrosion are: strong bands at 3445 and 3336 cm^−1^ representing N–H asymmetric and O–H stretching vibrations, a medium broad band centered around 1639 cm^−1^ encompassing N–H amide and C=C vibrations and a strong peak at 1030 cm^−1^ characteristic of silicates^39^. The absence of bands around 2900–2880 cm^−1^ indicate that long-chain aliphatic compounds may not be present or beyond the detection limit of the technique.

The presence of silicates in the interior corrosion of the Cauldron Rim was confirmed by XRD with the identification of quartz (Supplementary Fig. S1) in the sample. Quartz (SiO_2_) is a crystalline mineral present in UK soils as sand grains^40^. Whilst the hardness of this sample can be explained by the presence of quartz crystals, no evidence for its blue/greenish colour such as copper(II) minerals was found. The exterior corrosion sample from the Cauldron Rim did not yield a diffractogram.

**Figure 3:**
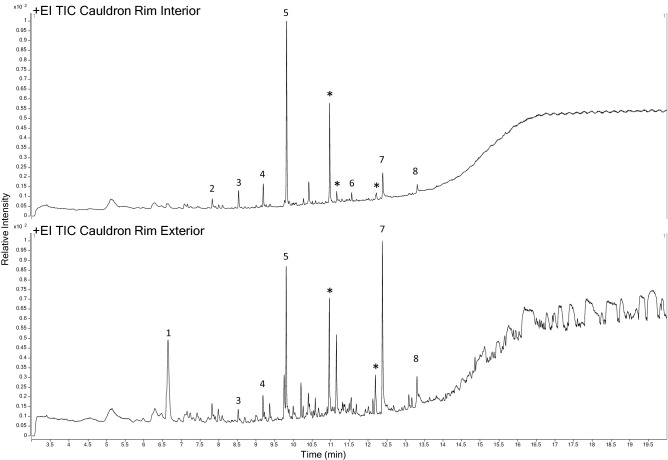
Total Ion Chromatograms obtained from the solid Cauldron Rim corrosion samples Compounds identified [1] Cymene; [2] Dodecene; [3] Tridecene; [4] Tetradecene; [5] Pentadecene; [6] Undecyl-benzene; [7] n-Hexadecanoic acid; [8] Octadecanoic acid and [*] long-chain aliphatic compounds. Spectral information is included in Supplementary Tables S1, 2.

The Total Ion Chromatograms obtained from the analysis of the corrosion from the interior and exterior cauldron rim (Fig. 3) were very similar. Both contain peaks identified as medium-chain n-alkenes [3, 4 and 5], two ubiquitous long-chain carboxylic acids [7 and 8] and long-chain aliphatic compounds. Most compound measurements, represented by unique *m/z* values, were detected in the exterior corrosion sample, which included a relatively high abundance peak matched to a plant metabolite aromatic hydrocarbon [1].

### Evidence of a unique corrosive microclimate detected in the exterior of Vessel A

**Figure 4:**
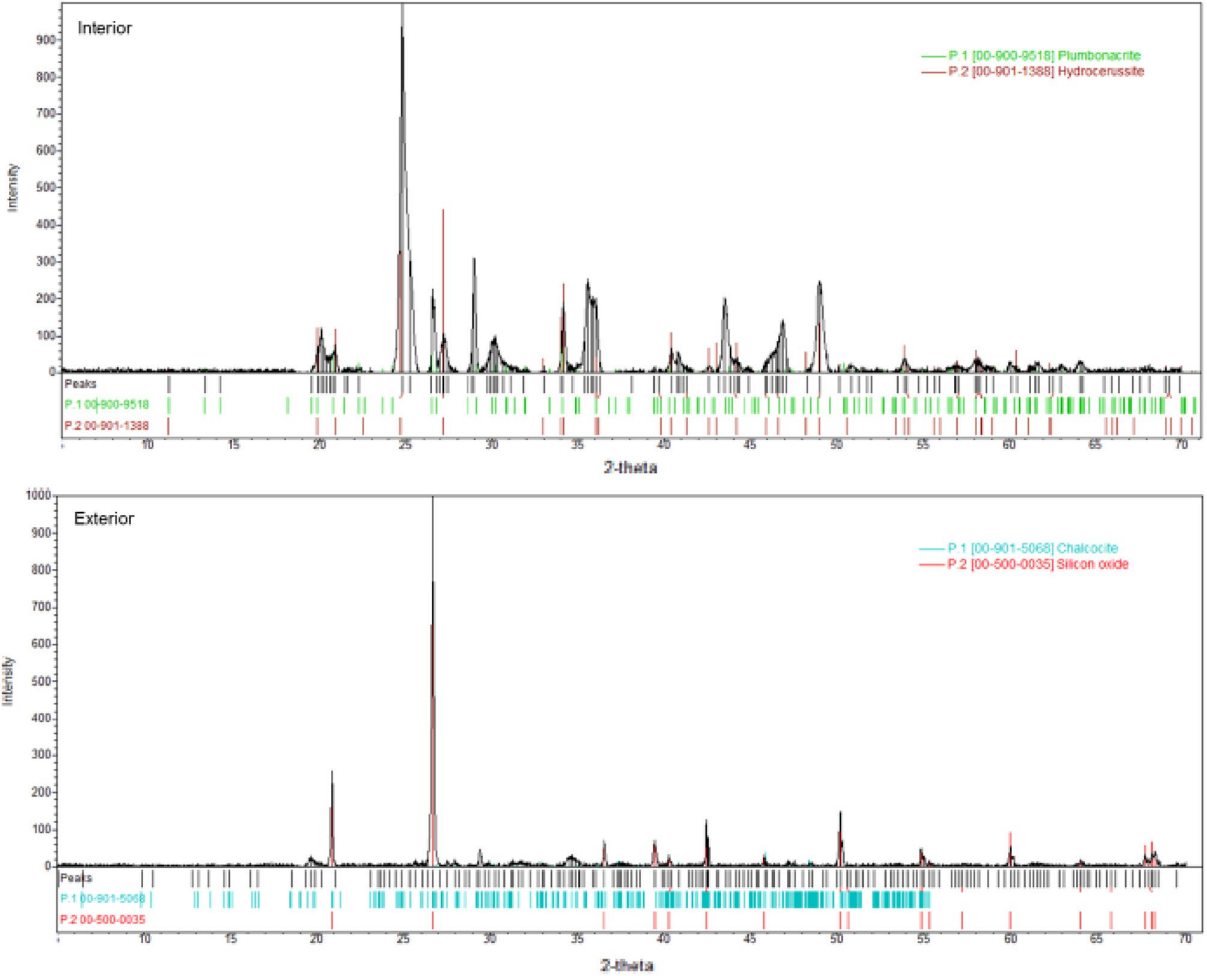
Diffractograms obtained by XRD for the Vessel A corrosion with phase identification plumbonacrite and hydrocerusside identified in the interior sample, chalcocite and silicon oxide (quartz) identified in the exterior sample.

The FTIR spectra of Vessel A corrosion samples (Supplementary Fig. S1) yielded only broad peaks that could not be assigned to any chemical function. By contrast, four mineral phases were identified in these samples by XRD (Fig. 4). The interior sample was characterized as 75% Pb_3_(CO_3_)_2_(OH)_2_ (hydrocerussite) and 25% Pb_10_(CO_3_)_6_O(OH)_6_ (plumbonacrite). The presence of these compounds in the sample may reflect the composition of the alloy used to make the vessel^41^ where lead corroded preferentially to copper^42^. Alternatively, the lead carbonates may relate to vessel use, given that in Roman times lead carbonate was used as make-up and added to medicinal preparations for the treatment of ulcers^43,44^.

The exterior corrosion of Vessel A was characterized as 86% quartz and 14% Cu_2_S (chalcocite). Cuprous sulfides (including chalcocite) are typically dark corrosion products found on archaeological copper alloy objects^45^ that have been exposed to sulfate-reducing bacteria in anaerobic conditions^46^.

**Figure 5:**
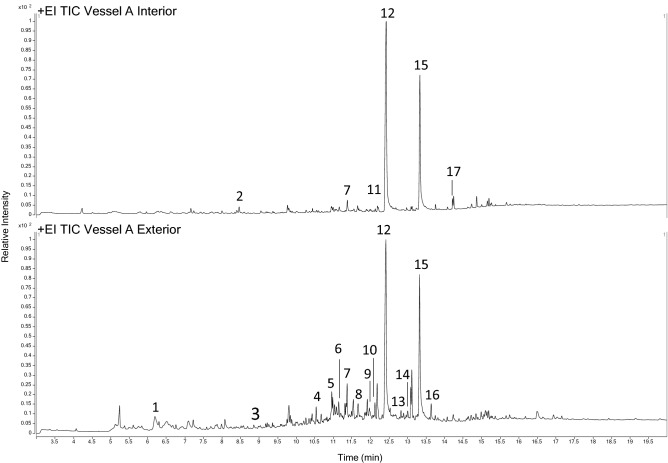
Total Ion Chromatograms obtained from the solid Vessel A corrosion samples.

The organic compounds recovered from these samples were also intriguing. The Total Ion Chromatograms obtained from the corrosion of Vessel A (Fig. 5) were dominated by two peaks identified as long-chain carboxylic acids [12 and 15]. However, polycyclic aromatic hydrocarbons [3, 5, 8, 9, 13, 16], phthalate [4] and long-chain aliphatic nitriles [10 and 14] were only identified in the exterior sample.

Compounds identified: [1] 5-methyl Furfural/benzaldehyde; [2] Methenamine; [3] 1-methyl Naphthalene; [4] Phthalate; [5] 2,6-Diisopropylnaphthalene; [6] Pentadecanal; [7] Tetradecanoic acid; [8] Fluorenone; [9] Phenanthrene; [10] aliphatic compound containing nitrile functional group; [11] Hexadecanoid acid methyl ester; [12] n-Hexadecanoic acid; [13] 2-phenyl Naphthalene; [14] 9-Octadecenitrile; [15] Octadecanoic acid; [16] Pyrene; [17] Octadecanoic acid, butyl ester. Spectral information is listed in Supplementary Tables S3, 4.

The origin of PAHs in the environment is often attributed to anthropogenic action (i.e. burning of wood^47^ and fossil fuels such as bitumen)^48,49^. Copper alloy vessels are reported to have been coated in bituminous substances to protect them from fire damage but their markers (i.e. a homologous n-alkanes series, hopanes, steranes and terpanes patterns)^50–52^ were either not present in the sample or are not detectable by the method used in this study. Additional analysis of the exterior corrosion sample targeting bitumen markers^53^ would be required to provide conclusive evidence of the absence of these markers.

PAHs can also have a biological source. These compounds have been detected in the tropical forest^54^, sediments^55^, soils^56^, in peatlands flora^57^, in *Magnolia* flowers^58^, termite nests^59^ (as a microbial metabolite and as a decomposition product of plant material) and as a fungus metabolite^60^. If we assume that the area between Vessel A and the cauldron originally contained plant remains as packing material (similar to the plant remains found under the scale plans in Vessel B), rainwater percolating through the soil would accumulate in this area facilitating its bacterial decay under an anaerobic environment. Such conditions could have led to the formation of PAHs^61^ and supported sulfate-reducing bacteria responsible for the chalcocite layer.

Analysis of the soil under Vessel B yielded a chromatogram (Fig. S3) dominated by hexadecanoic acid, an adipic acid ester, other minor compounds (including naphthalene), siloxanes but no compounds with the nitrile function. Long-chain alkyl nitriles are amongst the characteristic nitrogen-containing compounds of silty and clay soils^62^, occurring in nature as bacterial metabolites^63^, and detected as a pyrolytic product of archaeological soils rich in organic matter^64^. Thus the long-chain alkyl nitriles found in the exterior corrosion of Vessel A may indicate that this area of the hoard was once rich in organic matter.

### Potentially historical residues detected in the interior of Vessel B

The FTIR spectra of the corrosion samples from the interior of Vessel B (Supplementary Fig. S3) have only  broad non-diagnostic peaks. In spite of their greenish/brown colour, the only crystalline phase identified in these samples was quartz, although many peaks remained unassigned, especially for the sample from the base of the vessel.

**Figure 6:**
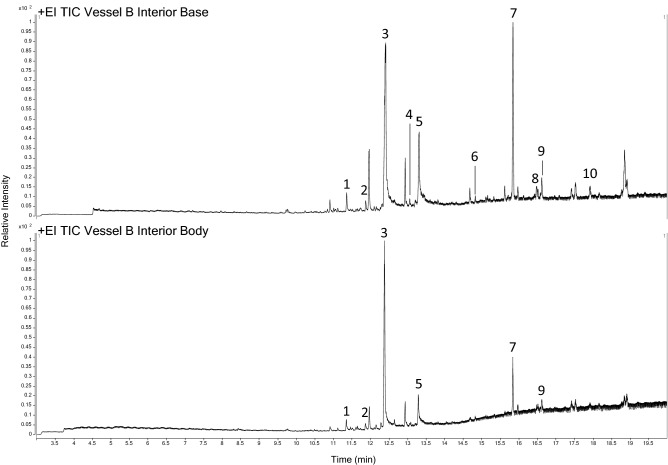
Total Ion Chromatograms obtained from the solid Vessel B corrosion samples Compounds identified: [1] Tetradecanoic acid; [2] Pentadecanoic acid; [3] n-Hexadecanoic acid; [4] aliphatic compound containing a nitrile functional group; [5] Octadecanoic acid; [6] Phthalate [7] Squalene; [8] compound containing a steroid nucleus; [9] Cholesta-3,5-diene; [10] Cholesterol. Spectral information is listed in Supplementary Table S3.

The Total Ion Chromatograms obtained from these samples were practically identical (Fig. 6). The compounds with the highest relative abundance peaks were identified as long-chain carboxylic acids [compounds 1–3 and 5], a compound normally associated with plasticizers [6], a triterpene [7], an animal sterol [10] and compounds containing a steroid nucleus [8 and 9].

The interior of Vessel B was the most protected area of the hoard, thus explaining the preservation of various plants remains. The most informative organic residue profile was obtained from the base/bottom sample of this vessel where squalene, cholesterol and its oxidation products were also identified.

Cholesterol is rarely found in organic residues extracted for archaeological ceramics^65^, possibly due to its degradation resulting from interactions with the fired clay and fatty acids, conditions created by cooking in clay pots^66^. Therefore, when cholesterol is identified in archaeological residues its source is considered to be modern contamination from fingerprints^67^, particularly when squalene is also present. However, cholesterol and its oxidation products have been identified in carbonized residues found in copper cauldrons^20 ^, where the presence of copper ions has been suggested to have contributed to their preservation. It is also relevant that only cholesterol (not its oxidation products) was identified in modern freeze-dried milk and copper-milk corrosion^33 ^analysed by the same GC-QTOF-MS with TSP method used in this study, and no sterols have been identified in any other sampls from the Pewsey Hoard.

## Conclusion

The adoption of a multi-analytical protocol for the characterization of the Pewsey Hoard corrosion enabled us to identify organic and inorganic phases. The initial assumption regarding analytical techniques was that that FTIR and powder XRD could be used as screening techniques for organic and inorganic phases respectively. FTIR spectra were non-diagnostic even after suspension of the samples in KBr. Analysis by powder XRD was also challenging because some samples were not sufficiently large to yield a diffractogram or did not contain crystalline phases other than quartz.

The main technique for targeting organic residues was GC-QTOF-MS using a thermal separation probe. The technique supports an untargeted approach to sample characterization with practically no sample preparation. The wide range of compounds identified in the corrosion of the Pewsey Hoard vessels appear to reflect microclimates reflecting how the hoard was assembled and included biomarkers for animal fat found in the most protected area of the hoard from contact with the deposition environment.

Contamination is a major factor that complicates organic residue analysis in archaeology. In the case of corrosion samples, potential sources of contaminants include the deposition environment (e.g. soil, water), handling of the object during and post-excavation, contact with packaging, conservation treatments applied to the object in the field and contamination introduced during processing of samples in the laboratory. The Pewsey Hoard was excavated and transported to a museum by the finders, who probably did not wear gloves. Although precautions against contamination (wearing disposable gloves, using disposable metal scalpels and sterilized glass vials) were taken when sampling the vessels, it is possible that the sampled surfaces had already been contaminated prior to the arrival of the objects at the museum. It is tempting to disregard the findings presented in this study because of potential contamination and the absence of controls. However we hope that our study will raise awareness of the potential of metal corrosion products for organic residue analysis, a material that is often removed and discarded or considered a simple mixture of inorganic compounds.

## Materials and methods

### Fourier-transform infrared (FTIR)

Samples were analysed in reflection or in transmission mode. In reflection mode, the technique used was Attenuated Total Reflectance (ATR) where around 1 mg of sample was pressed against the reflective crystal of the equipment. In transmission mode, the technique used was the KBr pellet method. The sample was mixed with KBr in a 2/200 mg ratio. The mixture was pressed to form a 1 mm thick disc for analysis. Measurements were taken with an Excalibur Series Varian UMA600 as a combination of 64 scans between the 4000–400 cm^−1^ range and included background subtraction. Data was processed with Digilab Resolutions Pro 4.0 software, figures created with Spectragryph v.1.2.12 and band assignments were based on FTIR tables^39,68^.

### Powder X-ray diffraction (XRD)

The pulverized samples were set on a single crystal silicon plate using a PANalytical X’Pert PRO Cu alpha instrument at the Crystallography Laboratory in the Department of Chemistry, University of Oxford. The equipment was set to operate in continuous mode at 40 kV/40 mA with a scanned area set between 1° and 70° 2θ, 0.02 step size and 3° per minute. Data was processed using QualX© software with phase identification using the Crystallography Open Database (COD).

### Gas chromatography with Qquadrupole time-of-flight mass spectrometry using a thermal separation probe (GCQTOF- MS with TSP)

A glass microvial containing 3 mg of sample was placed inside the TSP attached to an Agilent 7890B gas chromatograph equipped with a Restek Rxi-5 ms column (30 m × 320 μm × 0.25 μm). The mass spectrometer was an Agilent 7250 GC/Q‑TOF equipped with a low-energy-capable EI source (70 eV). The TSP was set at 300 °C and the oven temperature set at 40 °C for one minute, increasing by 20 °C/minute until 320 °C where it was held for five minutes. Helium was used as a carrier gas, at 1.43L/min flow rate and 8.7psi pressure. The equilibration time was 0.5 min and the sample injection was splitless. The mass range was 50 to 650 m*/*z. All samples were run in triplicate with blanks added between samples. Data analysis was performed using Agilent Mass Hunter Qualitative Analysis 10.0 with compound assignments using NIST Library 17. Only compounds with a match factor (MF) and reverse match factor (RMF) > 700 and where the molecular ion is present with an accuracy mass error below 50 ppm were shortlisted.

## Supplementary Information

Supplementary Information.

## Data Availability

The raw datasets used during the current study are available from the corresponding author on reasonable request.
