# Supplementary material for: Unlocking the organic residues preserved in the corrosion from the Pewsey Hoard vessels
Source: Sci Rep. 2022 Dec 9;12:21284. doi: 10.1038/s41598-022-24400-5 (PMC9734644; doi:10.1038/s41598-022-24400-5)
Supplement: Supplementary file 1 — Supplementary Information. [file 41598_2022_24400_MOESM1_ESM.docx]

**Unlocking the organic residues preserved in the corrosion from the Pewsey Hoard**

Luciana da C. Carvalho, Richard Henry, James McCullagh, A Mark Pollard

Luciana da C. Carvalho

Email: [luciana.carvalho@linacre.ox.ac.uk](mailto:xxxxx@xxxx.xxx)

**Supplementary Information**

[Figure S 1 - FTIR spectra of Vessel A samples obtained with KBr method 2](#_Toc113443743)

[Figure S 2 - FTIR spectra of Vessel B samples obtained with KBr method 2](#_Toc113443744)

[Figure S 3 - Total Ion Chromatograms obtained from soil under Vessel B 3](#_Toc113443745)

[Figure S 4 – Diffractograms obtained by XRD for the Vessel B corrosion with phase identification quartz is the main phase present and minor peaks remained unassigned in both samples. 3](#_Toc113443746)

[Table S 1 - Spectral information for compounds identified in the Cauldron Rim’s interior corrosion 4](#_Toc113443758)

[Table S 2 - Spectral information for compounds identified in the Cauldron Rim’s exterior corrosion 6](#_Toc113443759)

[Table S 3 - Spectral information for compounds identified in Vessel A’s interior corrosion 8](#_Toc113443760)

[Table S 4 - Spectral information for compounds identified in Vessel A’s exterior corrosion 9](#_Toc113443761)

[Table S 5 - Spectral information for compounds identified in soil under Vessel B 12](#_Toc113443762)

[Table S 6 - Spectral information for compounds identified in Vessel B’s interior (base/bottom) corrosion 14](#_Toc113443763)

[Table S 7 - Spectral information for compounds identified in Vessel B’s interior (body/side) corrosion 16](#_Toc113443764)


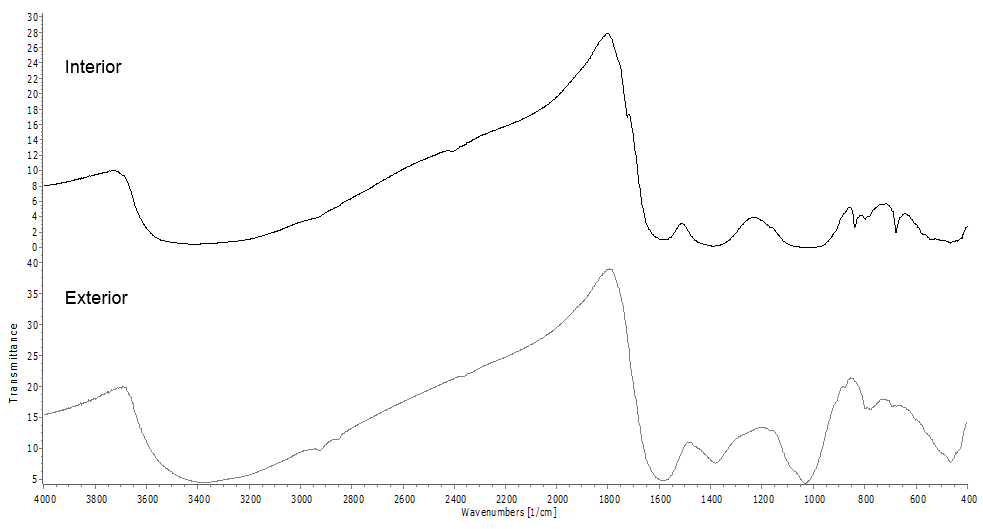


Figure S 1 - FTIR spectra of Vessel A samples obtained with KBr method


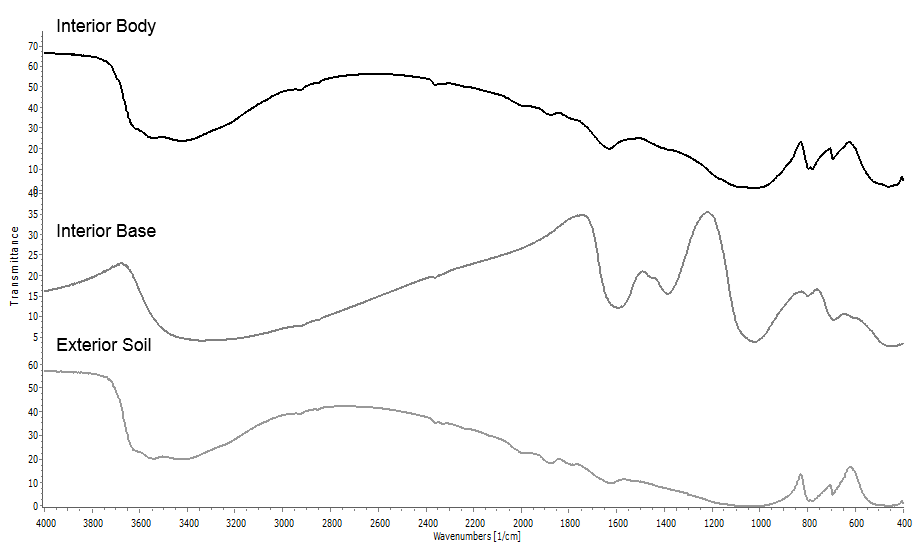


Figure S 2 - FTIR spectra of Vessel B samples obtained with KBr method


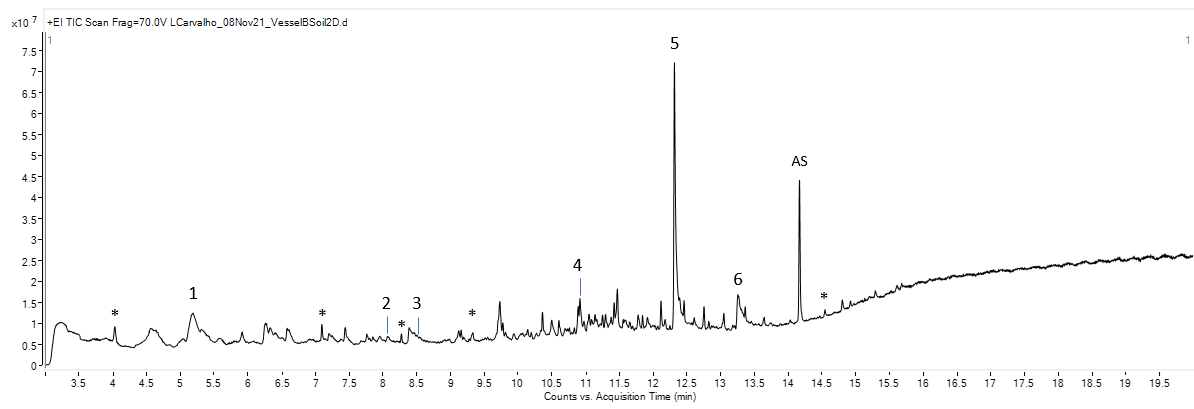


Figure S 3 - Total Ion Chromatograms obtained from soil under Vessel B

Compounds identified: [1] Furfural; [2] Naphthalene; [3] Methenamine; [4] Docosene; [5] n-Hexadecanoic acid; [6] Octadecanoic acid; [AS] adipic acid ester [*] siloxanes. Spectral information is listed in Supplementary Table S2.

**
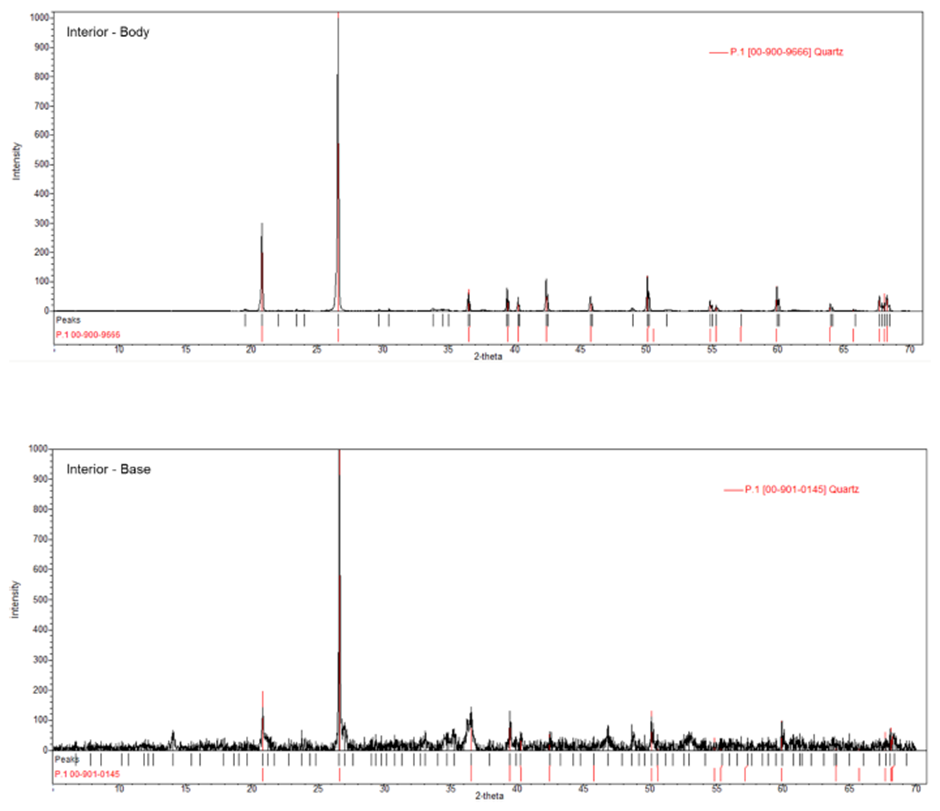
**

Figure S 4 – Diffractograms obtained by XRD for the Vessel B corrosion with phase identification quartz is the main phase present and minor peaks remained unassigned in both samples.

Table S 1 - Spectral information for compounds identified in the Cauldron Rim’s interior corrosion

| **PEAK 2** (7.83 min) **DODECENE** – MF863/RMF906 – Measured M-ion: 168.1805 *m/z -* Mass error: -43.40 ppm | | | | | | | |
| --- | --- | --- | --- | --- | --- | --- | --- |
|  | | | | | | | |
| **PEAK 3** (8.53 min) **TRIDECENE** – MF800/RMF811 – Measured M-ion: 182.1959 *m/z* - Mass error: -41.44 ppm | | | | | | | |
|  |  |  |  |  |  |  |  |
|  | | | | | | | |
| **PEAK 4** (9.19 min) **TETRADECENE** – MF868/RMF902 – Measured M-ion:: 196.2111 *m/z* – Mass error: -40.76 ppm | | | | | | | |
|  | | | | | | | |
| **PEAK 5** (9.82 min) **PENTADECENE** – MF893/RMF901 – Measured M-ion: 210.2267 *m/z* - Mass error: -38.30 ppm | | | | | | | |
|  | | | | | | | |
| **PEAK 6** (11.56 min) **UNDECYL-BENZENE** – MF787/RMF841 – Measured M-ion: 232.2099 *m/z* - Mass error: -39.62 ppm | | | | | | | |
|  | | | | | | | |
| **PEAK 7** (12.39 min) **N-HEXADECANOIC ACID*** – MF838/RMF871 – Measured M-ion: 256.2301 *m/z* - Mass Error: -39.53 ppm | | | | | | | |
|  | | | | | | | |
|  | | | | | | | |
| **PEAK 8** (13.31 min) **OCTADECANOIC ACID^*^** – MF724/RMF827 – Measured M-ion: 284.2590 *m/z* – Mass Error: -44.08 ppm | | | | | | | |
|  | | | | | | | |

*Confirmed with a standard

Head to Tail Spectra: unknown in RED (top) and assignment in BLUE (bottom)

Table S 2 - Spectral information for compounds identified in the Cauldron Rim’s exterior corrosion

| **PEAK 1** (6.64 min) | | | | **CYMENE** – MF762/RMF903 – Measured M-ion: 134.1038 *m/z* - Mass error: -42.86 ppm | | | | | |
| --- | --- | --- | --- | --- | --- | --- | --- | --- | --- |
|  | | | | | | | | |  |
| **PEAK 3** (8.52 min) | | | | **TRIDECENE** – MF708/RMF783 – Measured M-ion: 182.1942 *m/z* - Mass error: -50.77 ppm | | | | | |
|  | | | | | | | | |  |
| **PEAK 4** (9.18 min) | | | | **TETRADECENE** – MF780/RMF908 – Measured M-ion: 196.2084 *m/z* - Mass error: -54.54 ppm | | | | | |
|  | | | | | | | | |  |
| **PEAK 5** (9.81 min) | | | | **PENTADECENE** – MF870/RMF886 – Measured M-ion: 210.2251 *m/z* - Mass error: -45.91 ppm | | | | | |
|  | | | | | | | | |  |
| **PEAK 7** (12.38 min) **N-HEXADECANOIC ACID*** – MF875/RMF886 – Measured M-ion: 256.2291 *m/z* - Mass error: -43.58 ppm | | | | | | | | | |
|  | | | | | | | | |  |
| **PEAK 8** (13.30 min) **OCTADECANOIC ACID*** – MF752/RMF824 – Measured M-ion: 284.2597*m/z* - Mass error: -41.62 ppm | | | | | | | | | |
|  | | | | | | | | |  |
|  |  |  |  | |  |  |  |  |  |

*Confirmed with a standard

Head to Tail Spectra: unknown in RED (top) and assignment in BLUE (bottom)

Table S 3 - Spectral information for compounds identified in Vessel A’s interior corrosion

| **PEAK 2** (8.46 min) | | | | **METHENAMINE** – MF810/RMF901 – Measured M-ion: 140.1054 *m/z* - Mass error: -5.68ppm | | | | | |
| --- | --- | --- | --- | --- | --- | --- | --- | --- | --- |
|  | | | | | | | | |  |
| **PEAK 7** (11.37 min) **TETRADECANOIC ACID** – MF738/RMF918 – Measured M-ion: 228.2078 *m/z* - Mass error: -4.95 ppm | | | | | | | | | |
|  | | | | | | | | |  |
| **PEAK 11** (12.19 min) **HEXADECANOIC ACID METHYL ESTER** – MF803/RMF853 – Measured M-ion: 270.2555 *m/z -*  Mass error: -1.41 ppm | | | | | | | | | |
|  | | | | | | | | |  |
| **PEAK 12** (12.42 min) **n-HEXADECANOIC ACID*** – MF876/RMF885 – Measured M-ion: 256.2387 - Mass error: -5.97 ppm | | | | | | | | | |
|  | | | | | | | | |  |
| **PEAK 15** (13.33 min) **OCTADECANOIC ACID*** – MF857/RMF873 – Measured M-ion: 284.2697 *m/z* - Mass error: -6.09 ppm | | | | | | | | | |
|  | | | | | | | | |  |
|  |  |  |  | |  |  |  |  |  |

*Confirmed with a standard - Head to Tail Spectra: unknown in RED (top) and assignment in BLUE (bottom)

Table S 4 - Spectral information for compounds identified in Vessel A’s exterior corrosion

| **PEAK 1** (6.19 min) **5-METHYL FURFURAL** – MF635/RMF887 – Measured M-ion: 110.0288 *m/z*  - Mass error: -72.52 ppm | | |
| --- | --- | --- |
|  | | |
| **BENZALDEHYDE** – MF690/RMF867 – Measured M-ion: 106.0342 *m/z*  - Mass error: -72.28 ppm | |  |
|  | | |
| **PEAK 3** (8.86 min) **1-METHYL NAPHTHALENE** – MF646/RMF862 – Measured M-ion: 142.0720 *m/z*  - Mass error: -43.98 ppm | | |
|  | | |
| **PEAK 5** (11.02 min) **2,6-DIISOPROPYLNAPHTHALENE** – MF719/RMF804 – Measured M-ion: 212.1501 *m/z*  - Mass error: -30.17 ppm | | |
|  | | |
| **PEAK 6** (11.15 min) | **PENTADECANAL** – MF777/RMF912 – Measured M-ion: 226.2295 *m/z*  - Mass error: -0.73 ppm | |
|  | | |
| **PEAK 7** (11.38 min) **TETRADECANOIC ACID** – MF738/RMF888 – Measured M-ion: 228.2007 *m/z*  - Mass error: -36.06 ppm | | |
|  | | |
| **PEAK 8** (11.67 min) | **FLUORENONE** – MF811/RMF909 – Measured M-ion: 180.0531 *m/z -*  Mass error: -24.55 ppm | |
|  | | |
| **PEAK 9** (11.90 min) | **PHENANTHRENE** – MF738/RMF884 – Measured M-ion: 178.0736 *m/z -*  Mass error: -26.11 ppm | |
|  | | |
| **PEAK 12** (12.42 min) **n-HEXADECANOIC ACID*** – MF800/RMF857 – Measured M-ion: 256.2327 *m/z -*  Mass error: -29.39 ppm | | |
|  | | |
| **PEAK 13** (12.83 min) **2-PHENYL-NAPHTHALENE** – MF645/RMF902 – Measured M-ion: 204.0877 *m/z -* Mass error: -30.38 ppm | | |
|  | | |
| **PEAK 14** (13.00 min) **9-OCTADECENITRILE** – MF785/RMF865 – Measured M-ion: 263.2546 *m/z -* Mass error: -25.45 ppm | | |
|  | | |
| **PEAK 15** (13.34 min) **OCTADECANOIC ACID*** – MF711/RMF806 – Measured M-ion: 284.2641 *m/z -*  Mass error: -26.14 ppm | | |
|  | | |
| **PEAK 16** (13.64 min) | **PYRENE** – MF789/RMF840 – Measured M-ion: 202.0730 *m/z -* Mass error: -25.98 ppm | |
|  | | |
| \| **PEAK 17** (14.22 min) **OCTADECANOIC ACID, BUTYL ESTER** – MF870/RMF877 – Measured M-ion: 340.3324 *m/z -*  Mass error: -5.09 ppm \| \| --- \| \|  \| | | |

*Confirmed with a standard - Head to Tail Spectra: unknown in RED (top) and assignment in BLUE (bottom)

Table S 5 - Spectral information for compounds identified in soil under Vessel B

| **PEAK 1** (5.14 min) | | | **FURFURAL** – MF786/RMF868 – Measured M-ion: 96.0221 *m/z -* Mass error: 10.11 ppm | | | | | |
| --- | --- | --- | --- | --- | --- | --- | --- | --- |
|  | | | | | | | | |
| **PEAK 2** (8.06 min) | | | **NAPHTALENE** – MF745/RMF849 – Measured M-ion: 128.0645 *m/z -* Mass error: 14.87 ppm | | | | | |
|  | | | | | | | | |
| **PEAK 3** (8.50 min) | | | **METHENAMINE** – MF819/RMF851 – Measured M-ion: 140.1074 *m/z -* Mass error: 8.57 ppm | | | | | |
|  | | | | | | | | |
|  | | | | | | | | |
| **PEAK 4** (10.89 min) | | | **DOCOSENE** – MF819/RMF851 – Measured M-ion: 308.3447 *m/z -* Mass error: 1.29 ppm | | | | | |
|  | | | | | | | | |
|  | | | | | | | | |
| **PEAK 5** (12.32 min) | | | **n-HEXADECANOIC ACID*** – MF838/RMF846 – Measured M-ion: 256.2391 *m/z* Mass error: -4.41 ppm | | | | | |
|  | | | | | | | | |
|  | | | | | | | | |
| **PEAK 6** (13.25 min) | | | **OCTADECANOIC ACID*** – MF869/RMF897 – Measured M-ion: 284.2697 *m/z* Mass error: -6.44 ppm | | | | | |
|  | | | | | | | | |
|  |  |  | |  |  |  |  |  |

*Confirmed with a standard - Head to Tail Spectra: unknown in RED (top) and assignment in BLUE (bottom)

Table S 6 - Spectral information for compounds identified in Vessel B’s interior (base/bottom) corrosion

| **PEAK 1** (11.36 min) | | | **TETRADECANOIC ACID** – MF786/RMF868 – Measured M-ion: 228.2086 *m/z -* Mass error: -1.45 ppm | | | | | | |
| --- | --- | --- | --- | --- | --- | --- | --- | --- | --- |
|  | | | | | | | | | |
| **PEAK 2** (11.86 min) | | | **PENTADECANOIC ACID** – MF745/RMF849 – Measured M-ion: 242.2240 *m/z -* Mass error: -2.39 ppm | | | | | | |
|  | | | | | | | | | |
| **PEAK 3** (12.36 min) **n-HEXADECANOIC ACID*** – MF819/RMF851 – Measured M-ion: 256.2393 *m/z -* Mass error: -3.62 ppm | | | | | | | | | |
|  | | | | | | | | | |
|  | | | | | | | | | |
| **PEAK 4** (13.04 min) | | | **NITRILE(?)** | | | | |  | |
| 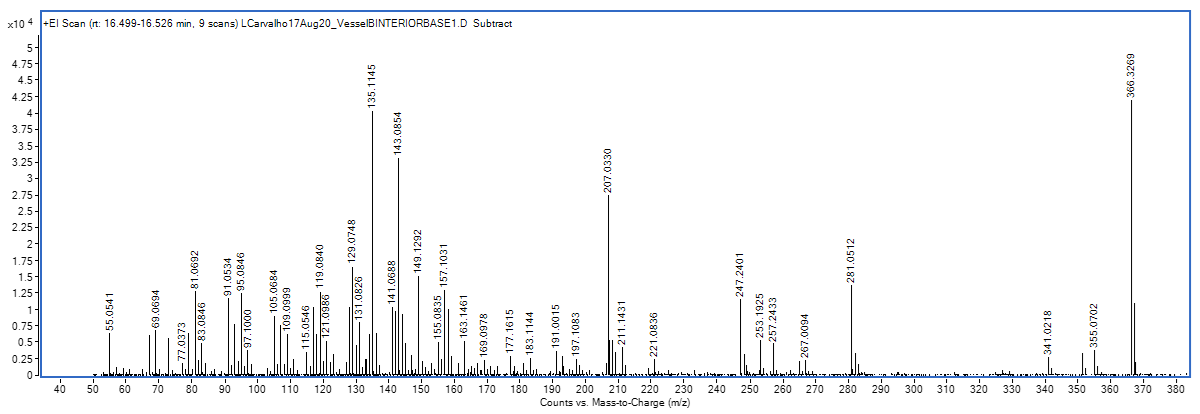 | | | | | | | | | |
|  | | | | | | | | | |
| **PEAK 5** (13.31 min) | | | **OCTADECANOIC ACID*** – MF838/RMF846 – Measured M-ion: 284.2714 *m/z* Mass error: -0.46 ppm | | | | | | |
|  | | | | | | | | | |
|  | | | | | | | | | |
| **PEAK 7** (15.85 min) | | | **SQUALENE** – MF869/RMF897 – Measured M-ion: 410.3910 *m/z* Mass error: -0.62 ppm | | | | | | |
|  | | | | | | | | | |
| **PEAK 9** (16.62 min) | | | **CHOLEST-3,5-DIENE** – MF860/RMF897 – Measured M-ion: 368.3421 *m/z -* Mass error: -5.98 ppm | | | | | | |
|  | | | | | | | | | |
| **PEAK 10** (17.92 min) | | | **CHOLESTEROL** – MF810/RMF885 – Measured M-ion: 368.3541 *m/z -* Mass error: -1.98 ppm | | | | | | |
|  | | | | | | | | | |
|  |  |  | |  |  |  |  | |  |

*Confirmed with a standard - Head to Tail Spectra: unknown in RED (top) and assignment in BLUE (bottom)

Table S 7 - Spectral information for compounds identified in Vessel B’s interior (body/side) corrosion

| **PEAK 1** (11.35 min) | | | **TETRADECANOIC ACID** – MF787/RMF866 – Measured M-ion: 228.2102 *m/z -* Mass error: 5.57 ppm | | | | | |  |
| --- | --- | --- | --- | --- | --- | --- | --- | --- | --- |
|  | | | | | | | | | |
| **PEAK 2** (11.86 min) | | | **PENTADECANOIC ACID** – MF748/RMF854 – Measured M-ion: 242.2251 *m/z -* Mass error: 2.15 ppm | | | | | |  |
|  | | | | | | | | | |
| **PEAK 3** (12.38 min) | | | **n-HEXADECANOIC ACID*** – MF840/RMF862 – Measured M-ion: 256.2411 *m/z -* Mass error: 3.40 ppm | | | | | |  |
|  | | | | | | | | | |
|  | | | | | | | | | |
| **PEAK 5** (13.29 min) | | | **OCTADECANOIC ACID*** – MF777/RMF826 – Measured M-ion: 284.2764 *m/z -* Mass error: -17.13 ppm | | | | | |  |
|  | | | | | | | | | |
| **PEAK 7** (15.84 min) | | | **SQUALENE** – MF845/RMF896 – Measured M-ion: 410.3975 *m/z -* Mass error: 15.22 ppm | | | | | |  |
|  | | | | | | | | | |
|  | | | | | | | | | |
|  | | | | | | | | | |
| **PEAK 9** (16.62 min) | | | **CHOLEST-3,5-DIENE** – MF726/RMF831 – Measured M-ion: 368.3491 *m/z -* Mass error: 13.03 ppm | | | | | |  |
|  | | | | | | | | | |
|  |  |  | |  |  |  |  |  | |

*Confirmed with a standard - Head to Tail Spectra: unknown in RED (top) and assignment in BLUE (bottom)
